# Allele-Selective Suppression of Mutant Huntingtin in Primary Human Blood Cells

**DOI:** 10.1038/srep46740

**Published:** 2017-04-24

**Authors:** James R. C. Miller, Edith L. Pfister, Wanzhao Liu, Ralph Andre, Ulrike Träger, Lori A. Kennington, Kimberly Lo, Sipke Dijkstra, Douglas Macdonald, Gary Ostroff, Neil Aronin, Sarah J. Tabrizi

**Affiliations:** 1 Department of Neurodegenerative Disease, UCL Institute of Neurology, University College London, London, WC1N 3BG, UK; 2 RNA Therapeutics Institute and Department of Medicine, University of Massachusetts Medical School, Worcester, MA, USA; 3 Charles River, Leiden, The Netherlands; 4 CHDI Management/CHDI Foundation, Los Angeles, CA, USA; 5 Program in Molecular Medicine, University of Massachusetts Medical School, Worcester, MA, USA

## Abstract

Post-transcriptional gene silencing is a promising therapy for the monogenic, autosomal dominant, Huntington’s disease (HD). However, wild-type huntingtin (HTT) has important cellular functions, so the ideal strategy would selectively lower mutant HTT while sparing wild-type. HD patients were genotyped for heterozygosity at three SNP sites, before phasing each SNP allele to wild-type or mutant *HTT*. Primary *ex vivo* myeloid cells were isolated from heterozygous patients and transfected with SNP-targeted siRNA, using glucan particles taken up by phagocytosis. Highly selective mRNA knockdown was achieved when targeting each allele of rs362331 in exon 50 of the *HTT* transcript; this selectivity was also present on protein studies. However, similar selectivity was not observed when targeting rs362273 or rs362307. Furthermore, HD myeloid cells are hyper-reactive compared to control. Allele-selective suppression of either wild-type or mutant HTT produced a significant, equivalent reduction in the cytokine response of HD myeloid cells to LPS, suggesting that wild-type HTT has a novel immune function. We demonstrate a sequential therapeutic process comprising genotyping and mutant *HTT*-linkage of SNPs, followed by personalised allele-selective suppression in a small patient cohort. We further show that allele-selectivity in *ex vivo* patient cells is highly SNP-dependent, with implications for clinical trial target selection.

Huntington’s disease (HD) is a fatal, autosomal dominant neurodegenerative disorder caused by a CAG repeat expansion in the huntingtin (*HTT*) gene^1^. Production of the mutant (m)HTT protein is the primary cause of pathology, and numerous studies have demonstrated that lowering cellular levels of mHTT using siRNA or antisense oligonucleotides is beneficial in animal models^2^,^3^,^4^. Most studies have utilised non-selective suppression of both mHTT and wild-type HTT, whereby the target nucleotide sequence is present on both *HTT* alleles. However, wild-type HTT has important roles in cellular function^1^, and although preclinical data suggests that partial lowering of wild-type HTT is tolerated in the adult brain^4^,^5^,^6^, intuitively the optimal strategy would selectively lower mHTT while sparing wild-type.

Allele-selective suppression may be achieved by genotyping HD patients to identify heterozygous single nucleotide polymorphisms (SNPs) in the *HTT* gene, before delivering siRNA specific for the SNP allele that is m*HTT*-associated^7^. Genotyping studies have suggested that 75% of Caucasian HD gene carriers would be treatable with five siRNAs targeting three SNPs^8^, and SNP-targeting strategies have been used successfully in human fibroblast and stem cell lines, as well as in transgenic mouse models of HD^9^,^10^,^11^. However, allele-selective suppression of mHTT has not yet been demonstrated in easily accessible primary *ex vivo* HD patient cells, with samples taken from a cohort of patients whose genotype was not known *a priori*. Carrying out a sequential workflow comprising personalised genotyping and mHTT suppression is key to determining the practical relevance of this approach, and has relevance to future potential HD clinical trials aimed at lowering mHTT.

## Results

### Allele-selective *HTT* suppression in HD patient cells using siRNA targeted to rs362331

We utilised siRNAs designed against the SNPs rs362331 in exon 50 (39.4% heterozygous C/T), rs362273 in exon 57 (35.2% heterozygous A/G) and rs362307 in the 3′-UTR (48.6% heterozygous C/T) of the *HTT* transcript. The siRNAs targeted to rs362273 and rs362307 have previously demonstrated good selectivity using luciferase assays in HeLa cells^8^. However, the siRNAs designed against rs362331 have not been previously published. As the T allele of rs362307 is linked to m*HTT*  8, testing siRNA targeting the C allele was unnecessary. Diagnostic DNA samples were analysed to determine individual SNP genotypes for a cohort of HD patients; we identified fifty-seven individuals who were heterozygous for rs362331, thirty-eight who were heterozygous for rs362273 and fifty-four who were heterozygous for rs362307. Monocyte-derived macrophages were then isolated from repeat peripheral blood samples donated by a subset of the heterozygous individuals. Transfection was carried out using glucan-encapsulated siRNA particles (GeRPs), which are taken up by phagocytosis before releasing their siRNA contents into the cytoplasm of the target cell. Cultures were harvested 72 h after transfection and mRNA expression was analysed by allele-specific qPCR. Previously validated non-selective anti-total *HTT* siRNA was used as a positive control^12^.

**Figure 1:**
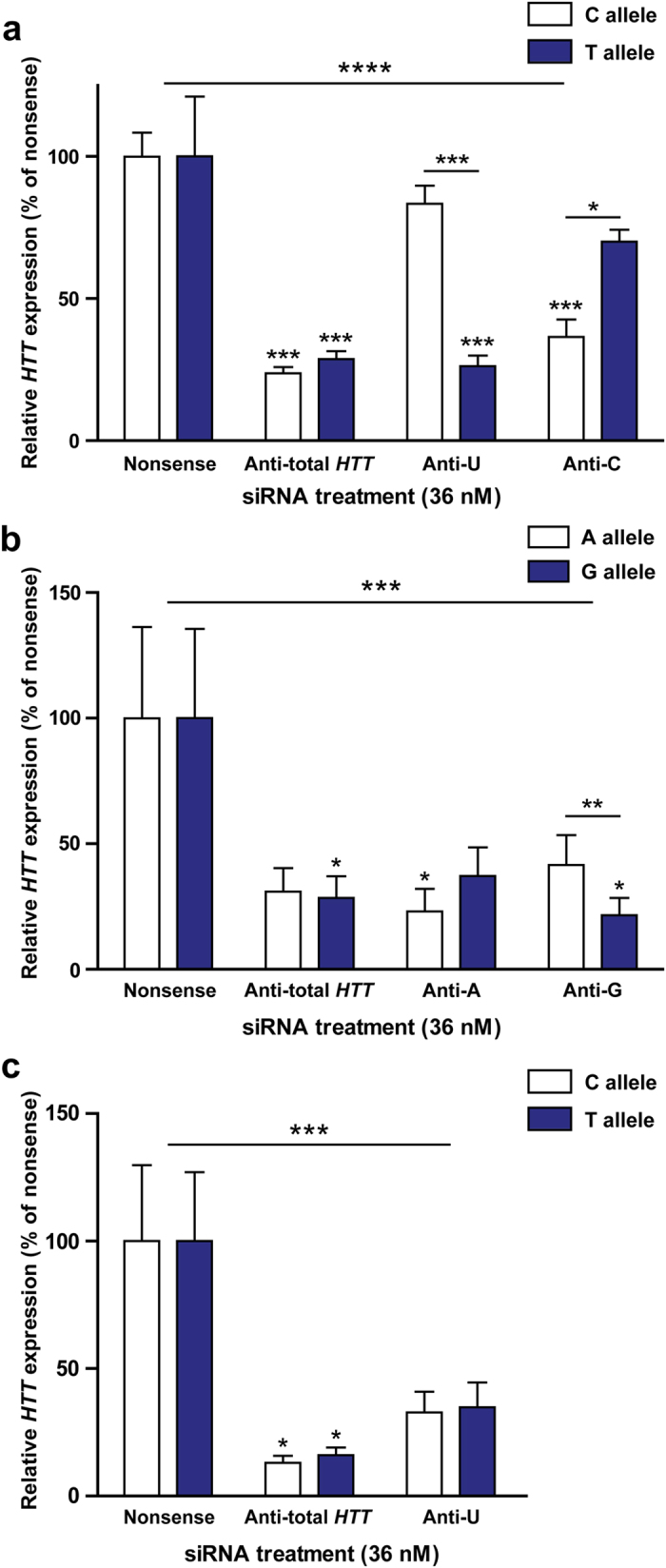
Characterisation of allele-selective siRNAs targeted to three SNPs in heterozygous human HD patient cells. Monocyte-derived macrophages were isolated from SNP-genotyped HD patients and treated with GeRPs containing either nonsense, anti-total *HTT* or allele-selective siRNA targeted to (**a**) rs362331 in exon 50, (**b**) rs362273 in exon 57 or (**c**) rs362307 in the 3′-UTR of the *HTT* transcript. Expression of each SNP allele was then measured 72 h later by qPCR. Data show mean expression of each SNP allele ± SEM (*n* = 3 for rs362331, *n* = 4 for rs362273 and rs362307), statistical analysis carried out using two-way ANOVA with Bonferroni post-tests. Asterisks above the horizontal line at the top of each graph show significance on ANOVA across the whole experiment before post-hoc testing. Asterisks above individual bars represent post-test significance for the mRNA expression of that allele compared to the same allele in the nonsense-treated samples. Asterisks above horizontal lines between two bars denote allele expression differences within the same treatment on post-hoc testing. *P < 0.05, **P < 0.01, ***P < 0.001, ****P < 0.0001. N = individual biological repeats.

Targeting rs362331 with anti-U siRNA resulted in 74% on-target U allele suppression, compared to 17% off-target C allele suppression (Fig. 1a). Targeting this SNP with anti-C siRNA resulted in 63% on-target suppression, with 30% off-target suppression. Post-hoc testing revealed the discrimination between alleles to be significant for each siRNA (p < 0.05). Indeed, in both cases on-target knockdown was equivalent to non-selective siRNA, while off-target knockdown was not statistically significant compared to nonsense control.

Unfortunately, comparable selectivity was not achieved when testing siRNAs targeted to either rs362273 or rs362307 (Fig. 1b,c). Although post-hoc testing demonstrated statistically significant allelic discrimination following treatment with the rs362273 anti-G siRNA, mean off-target knockdown of this degree is clearly too great to consider the particular siRNAs to be truly selective. These data are consistent with the poor discrimination previously seen when testing siRNA against rs362307 in cell lines and animal models^11^,^13^.

### Selective suppression of mHTT protein in HD patient cells using siRNA targeted to rs362331

**Figure 2:**
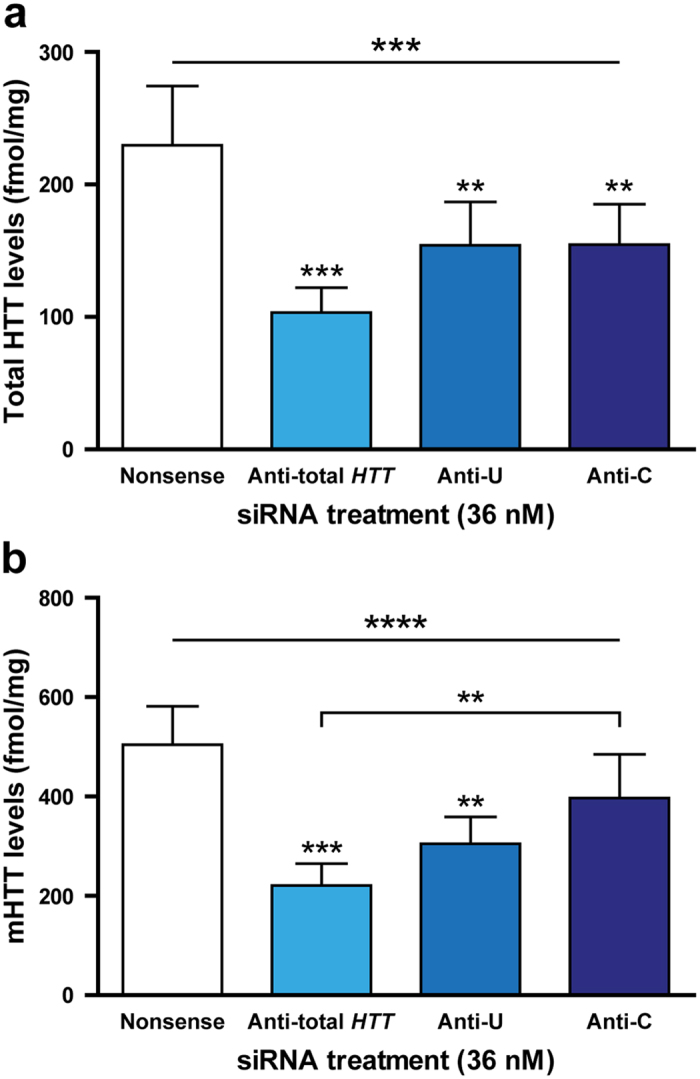
Selective suppression of mHTT protein in human HD patient cells using siRNA targeted to rs362331. Monocyte-derived macrophages were isolated from HD patients heterozygous for rs362331 with linkage of the T allele to m*HTT*, before treating with GeRPs containing either nonsense, anti-total *HTT* or allele-selective siRNA targeted to each allele of rs362331. Expression of (**a**) total and (**b**) mutant HTT protein was measured after 72 h using MSD assays, before normalisation to total cellular protein content as measured by BCA assays. Data show mean protein levels ± SEM (*n* = 5), statistical analysis carried out using one-way ANOVA with Tukey post-hoc testing. Asterisks above the horizontal line at the top of each graph show significance on ANOVA across the whole experiment before post-hoc testing. Asterisks above bars show post-test significance for each siRNA compared to the nonsense-treated samples. *P < 0.05, **P < 0.01, ***P < 0.001, ****P < 0.0001. N = individual biological repeats.

We next validated that total (polyQ-independent) and mHTT (polyQ-expanded) protein levels were similarly reduced; analysis was again carried out 72 h after transfection with siRNAs targeted to either allele of rs362331. As the siRNAs targeted to rs362273 and rs362307 did not achieve significant mRNA selectivity, it was decided not to advance their use to protein studies. To determine which siRNA was targeting m*HTT*, each patient’s SNP alleles were linked to either wild-type or m*HTT* using the SNP linkage by circularization technique^12^; the T allele of rs362331 was located on the m*HTT* allele in each subject used for protein analysis. As expected, we observed equivalent suppression of total HTT following treatment with each allele-selective siRNA, demonstrating that their effects on total HTT levels are independent of m*HTT*-linkage (Fig. 2a). However, we only observed significant suppression of mHTT compared to nonsense control with m*HTT*-targeted anti-U siRNA, with no significant reduction in mHTT following treatment with wild-type targeted anti-C siRNA (Fig. 2b). This result shows that the selectivity seen when targeting mRNA at rs362331 also affects the protein level.

### Allele-selective suppression of wild-type and mHTT reverses the hyper-reactive phenotype of human HD myeloid cells

**Figure 3:**
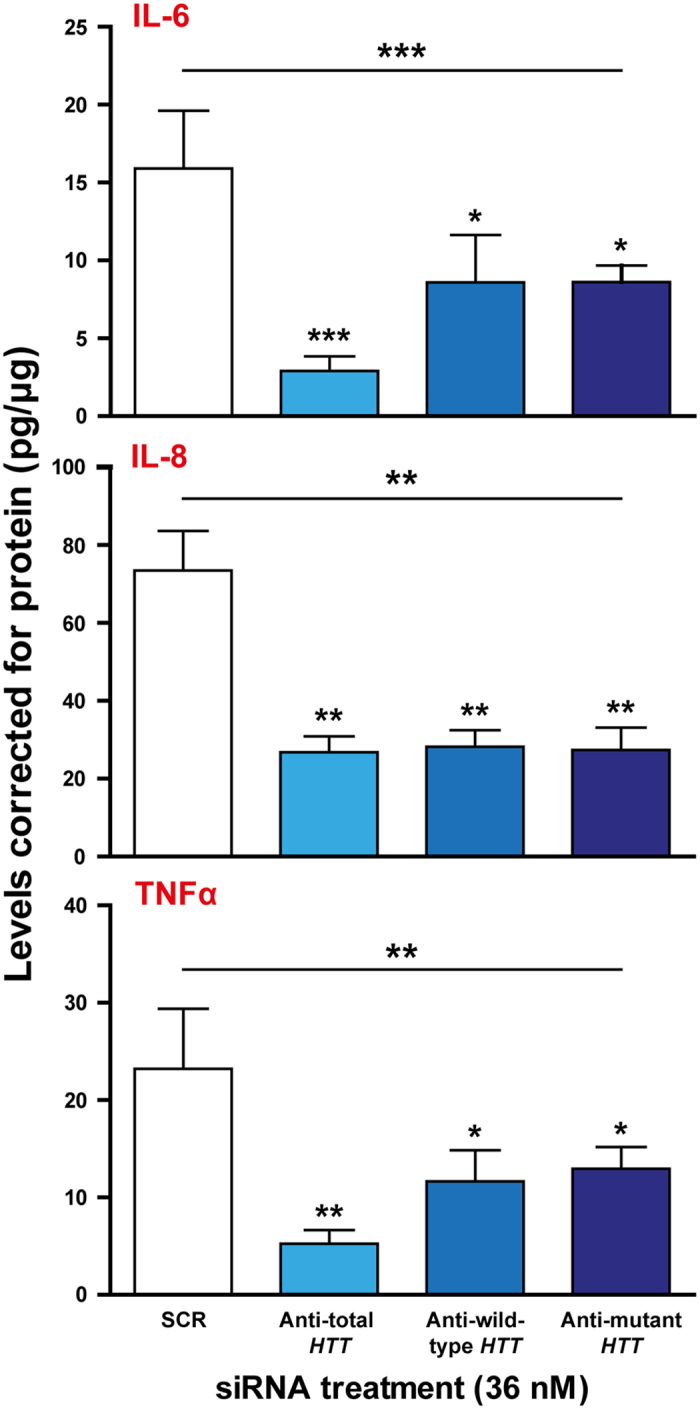
Allele-selective suppression of wild-type and mHTT reverses the hyper-reactive phenotype of human HD myeloid cells. Monocyte-derived macrophages were isolated from SNP-genotyped HD patients and treated with GeRPs containing either nonsense, anti-total *HTT* or allele-selective siRNA targeted to each allele of rs362331. Linkage of each SNP allele to either wild-type or m*HTT* was determined using the SNP linkage by circularization protocol. After 72 h the macrophages were stimulated with LPS and IFNγ, before the culture supernatants were collected after a further 24 h and analysed using MSD assays. Cytokine values were normalised to total protein content as measured by BCA assays. Data show mean cytokine levels ± SEM (*n* = 5 for IL-6 and TNFα, *n* = 4 for IL-8), statistical analysis carried out using one-way ANOVA with Tukey post-hoc testing. Asterisks above the horizontal line at the top of each graph show significance on ANOVA across the whole experiment before post-hoc testing. Asterisks above bars show post-test significance for each siRNA compared to the nonsense-treated samples. *P < 0.05, **P < 0.01, ***P < 0.001. N = individual biological repeats.

Previous work has shown that HD myeloid cells are hyper-reactive compared to control cells in response to stimulation with LPS and IFNγ^14^. This phenotype was found to be reversible following non-selective HTT suppression with siRNA. To address whether allele-selective suppression has similar effects, we stimulated monocyte-derived macrophages with LPS and IFNγ 72 h after treatment with allele-selective GeRPs targeting either allele of rs362331. Production of IL-6, IL-8 and TNFα was measured 24 h later. Since we observed no significant differences between the mRNA knockdown provided by the anti-U and anti-C siRNAs, the samples were grouped for analysis into anti-wild-type and anti-mHTT following SNP linkage to m*HTT*. Interestingly, allele-selective suppression of either wild-type or mHTT produced a significant reduction in the levels of each cytokine compared to nonsense control (Fig. 3). No differences in cytokine production were found when selectively suppressing mutant compared to wild-type HTT.

## Discussion

We demonstrate the first example of a complete sequential workflow comprising SNP genotyping, SNP linkage, and personalised allele-selective suppression with phenotypic reversal of an HD-related cellular deficit in heterozygous primary *ex vivo* patient cells. We achieved this through repeated sampling of a small patient cohort, using siRNA targeted to rs362331 in exon 50 of the *HTT* gene. These genotyping techniques would form the basis of allele-selective treatment in the clinic, and our study demonstrates their first use in combination, instead of individually as part of a technical study^8^,^15^. Indeed, blood cells are likely to be used for genotyping in clinical trials due to their easy accessibility, and provide a convenient screening tool for novel therapeutics. Although we saw reduced suppression of protein relative to mRNA, we consider this is likely due to the longer protein half-life allowing more pre-existing species to persist up to 72 h.

These data reinforce previous studies demonstrating that the potency and selectivity of m*HTT* suppression varies considerably depending on the SNP being targeted^9^,^11^. While we observed significant allele-selectivity using siRNAs targeted to both alleles of rs362331, we did not see similar effects when targeting either rs362273 or rs362307; this is likely due to additional factors including the nucleotide sequence and tertiary structure of the transcript surrounding the base mismatch. rs362307 is a particularly challenging target for allele-selective suppression as the surrounding sequence is extremely GC rich, with the SNP comprising a pyrimidine mismatch (purine mismatches typically confer greater selectivity)^7^. Indeed, this SNP has now demonstrated poor selectivity across multiple research groups and studies^11^,^13^. While previous work has shown that adding an additional base mismatch in the siRNA sequence can improve selectivity (often at the cost of reduced potency)^8^, the results seen with our anti-rs362307 siRNA suggest that the benefits of this approach may be limited. These data further demonstrate the importance of testing potential therapeutics in primary patient cells containing endogenous expression of full-length *HTT*, as these siRNAs achieved promising selectivity during preliminary testing with a short construct attached to a luciferase reporter^8^.

We further utilised allele-selective suppression to investigate the cytokine response of HD myeloid cells. HD myeloid cells produce increased levels of proinflammatory cytokines compared to control following stimulation with LPS^14^. This phenotype is due to an exaggerated NFκB signalling response caused by a direct interaction of mHTT with IKK, a key cytoplasmic regulator of NFκB^16^. NFκB signalling has recently been shown to be upregulated even in resting HD myeloid cells, and contributes to an abnormal proinflammatory transcriptional profile in the absence of any stimulation^17^. However, very little is known about whether wild-type HTT also has a role in innate immune function. If HTT’s effects on cytokine production were limited exclusively to the mutant form, it would be expected that selective mHTT-lowering would reduce cytokine production more than selective wild-type lowering. However, we saw no significant differences in cytokine production between the allele-selective treatment groups. These results suggest that wild-type HTT has an as yet undefined role in normal immune cell function, and is consistent with previous work showing that cytokine production by control myeloid cells is also reduced following HTT-lowering^14^. Although none of the differences reached statistical significance, the mean IL-6 and TNFα levels measured following total HTT-lowering were less than 50% of those measured following selective lowering of either the mutant or wild-type allele. This result suggests that total cellular HTT load may be an important factor influencing cytokine production, as the sparing of one allele by the allele-selective siRNAs will result in less overall knockdown than that achieved by anti-total *HTT* siRNA. However, a full investigation of this hypothesis is beyond the scope of this study.

Finally, as rs362307 is the most prevalent SNP in Caucasian HD patients with 48.6% heterozygosity^8^, it should not be assumed that the optimal SNPs will be targetable when estimating what percentage of HD patients may benefit from allele-selective therapeutics. As a result, it may be necessary to target less prevalent SNPs to improve selectivity, with a subsequent reduction in the treatable proportion of the HD patient population. Non-selective therapeutics are therefore likely to be used to treat the majority of adult HD patients, as partial HTT-lowering has been shown to be well tolerated in the mature brain^4^,^5^. However, the deleterious effects associated with *HTT* knockout in the developing brain^6^ suggest that an allele-selective strategy may be more useful for treating presymptomatic young adults. We demonstrate that rs362331 may be a promising candidate for this approach.

## Methods

### Collection and genotyping of human samples

Experiments were performed in accordance with the Declaration of Helsinki and approved by the University College London (UCL)/UCL Hospitals Joint Research Ethics Committee (REC 03/N008). All subjects provided informed written consent. SNP genotyping was carried out by amplifying the SNP with FlashTaq 2X Master Mix, before sequencing by GeneWiz, Inc. (South Plainfield, NJ). Primer sequences for amplification were as follows: rs362331 forward: GGGCATTCTGTGACTCGGTA; rs362331 reverse: GATAGGAACCCACCGTTCAT; rs362273 forward: AGTGACAAATCCCCAAGACC; rs362273 reverse: GAGCTTTTCTCCTGGGTGTG; rs362307 forward: GCTCTGCTCGCTCTCCAG; rs362307 reverse: GCAGAGACACGCACGTTG. SNP linkage to m*HTT* was performed using the SNP linkage by circularization technique, using previously published methodology^15^. Details of the subject cohort used for each experiment are contained in Supplementary Table S1.

### HTT suppression

GeRPs were synthesised and loaded with siRNA according to previously published methodology^18^. siRNA guide sequences were as follows: nonsense: 5′-pUUUCGAAGUACUCAGCGUGAG-3′; anti-total *HTT:* 5′-pUUCAUCAGCUUUUCCAGGGUC-3′; rs362331 anti-C: 5′-pUUACACAGUGGAUGAGGGAGC-3′; rs362331 anti-U: 5′-pGUACACAGUAGAUGAGGGAGC-3′; rs362273 anti-A: 5′-pUUUGAUUUGUAGCAGCAGCUU-3′; rs362273 anti-G: 5′-pUUUGCUCUGCAGCAGCAGCUU-3′; rs362307 anti-U: 5′-pCACACGGGCACAGACUUCCAA-3′. The siRNA Sequence Probability-of-Off-Targeting Reduction (siSPOTR) algorithm (https://sispotr.icts.uiowa.edu/sispotr/index.html) was used to identify additional transcripts with the potential for off-target suppression. Genes with an individual transcript Probability of Off-Target Score (tPOTS) ≥ 0.2 were then analysed using the GO annotation enrichment analysis tool (http://www.geneontology.org/) to identify overlapping biological processes. A complete list of siSPOTR results and GO pathways for the rs362331 siRNAs is contained in Supplementary Data S1.

To test the siRNAs, monocytes were isolated from peripheral blood samples by density centrifugation and magnetic cell sorting, before differentiation into macrophages as previously described^14^. Transfection was carried out on day three of the differentiation protocol by changing to fresh media containing GeRPs at a 10:1 particle to cell ratio. GeRPs were removed after 24 h by complete media change; this protocol achieves 90% transfection efficiency. Huntingtin mRNA and protein levels were then measured after a further 48 h in culture.

### Allele-specific qPCR

Custom TaqMan^®^ probes were designed for each SNP allele, before TaqMan^®^ Gene Expression Master Mix was used to analyse allele-specific *HTT* expression. Primers used for allele-specific qPCR were as follows: rs362331 forward: CTGGAGCGTGGTCTCCTCCACA; rs362331 reverse: GTGTGTTTGGATCTACTTCCTCC; rs362273 forward: CTACTACAGGTGCCCTCATCAG; rs362273 reverse: GTGACGAAGGTGCAGGGGCGTC; rs362307 forward: ATGGTGGGAGAGACTGTGAGGC; rs362307 reverse: ATGGCAGAGACACGCACGTTGC. Probes used for allele-specific qPCR were as follows: rs362331 C: 6FAM-TCCCTCATCCACTGTGTGC-MGBNFQ; rs362331 T: VIC-TCCCTCATCTACTGTGTGC-MGBNFQ; rs362273 A: 6FAM-GCTGCTGCTACAGATCAAC-MGBNFQ; rs362273 G: VIC-GCTGCTGCTGCAGATCAAC-MGBNFQ; rs362307 C: 6FAM-TGGAAGTCTGCGCCCTTGTG-MGBNFQ; rs362307 U: VIC-TGGAAGTCTGTGCCCTTGTG-MGBNFQ. Primers and probes were used at final concentrations of 500 nM and 150 nM respectively. Thermal cycling conditions were as follows: 50 °C for 2 min and 95 °C for 10 min followed by forty cycles of 95 °C for 15 s and either 65 °C (for rs362331 and rs362273) or 67.5 °C (for rs362307) for 1 min. *GAPDH* and *ACTB* were used as reference genes.

### HTT protein quantification assays

Total (polyQ-independent) and mHTT (polyQ-dependent) protein levels were measured by Meso Scale Discovery (MSD) assays using a protocol adapted from published methods^19^. 2B7 and biotinylated-4C9 were used as the antibody pair for detection of total HTT^20^, while 2B7 and biotinylated MW1 were used as the antibody pair for detection of mHTT^21^. Readings were normalised to total protein content of the sample as measured by BCA assays.

### MSD cytokine assays

Monocyte-derived macrophages were stimulated by changing to fresh media containing 2 μg/ml LPS and 10 ng/ml IFNγ 72 h after GeRP treatment. After a further 24 h the supernatants were analysed using the V-PLEX Human Proinflammatory Panel II (4-Plex) Kit. IL-1β was not included in the analysis as previous work has shown that its production is not upregulated in HD myeloid cells^14^. Readings were normalised to total protein content of the sample as measured by BCA assays.

### Statistical analysis

Statistical analysis was carried out using GraphPad Prism 6 (GraphPad). Analysis of mRNA knockdown was performed using two-way ANOVAs with Bonferroni post-hoc multiple comparison testing. Protein knockdown and cytokine profiling experiments were analysed using one-way ANOVAs with Tukey post-hoc multiple comparison testing. All error bars represent standard error of the mean.

## Additional Information

**How to cite this article:** Miller, J. R. C. *et al*. Allele-Selective Suppression of Mutant Huntingtin in Primary Human Blood Cells. *Sci. Rep.* **7**, 46740; doi: 10.1038/srep46740 (2017).

**Publisher's note:** Springer Nature remains neutral with regard to jurisdictional claims in published maps and institutional affiliations.

## Supplementary Material

Supplementary Table S1

Supplementary Data S1
