# Supplementary material for: Allele-Selective Suppression of Mutant Huntingtin in Primary Human Blood Cells
Source: Sci Rep. 2017 Apr 24;7:46740. doi: 10.1038/srep46740 (PMC5402279; doi:10.1038/srep46740)
Supplement: Supplementary Table S1 [file srep46740-s1.pdf]

Allele-Selective Suppression of Mutant Huntingtin in Primary Human Blood Cells

James RC Miller, Edith L Pfister, Wanzhao Liu, Ralph Andre, Ulrike Träger, Lori A Kennington, Kimberly Lo, Sipke Dijkstra, Douglas Macdonald, Gary Ostroff, Neil Aronin and Sarah J Tabrizi

Supplementary Table S1. Mean age and *HTT* CAG repeat length for subjects who donated samples for the experiments presented in the manuscript. Cohorts listed separately for each experiment.

Experiment	*n*	Age (mean ± SD)	CAG (mean ± SD)
mRNA expression targeting rs362331 (Fig. 1a)	3	50.1 ± 10.3	44.0 ± 2.2
mRNA expression targeting rs362273 (Fig. 1b)	4	51.2 ± 13.3	42.8 ± 0.8
mRNA expression targeting rs362307 (Fig. 1c)	4	55.9 ± 10.2	43.3 ± 1.5
Protein expression targeting rs362331 (Fig. 2)	5	51.1 ± 16.3	44.6 ± 2.7
Cytokine analysis of IL-6 and TNFα (Fig. 3)	5	49.9 ± 11.1	45.2 ± 2.6
Cytokine analysis of IL-8 (Fig. 3)	4	46.2 ± 9.3	46.0 ± 2.2
